# Accuracy of Prehospital Triage of Adult Patients With Traumatic Injuries Following Implementation of a Trauma Triage Intervention

**DOI:** 10.1001/jamanetworkopen.2023.6805

**Published:** 2023-04-04

**Authors:** Robin D. Lokerman, Eveline A. J. van Rein, Job F. Waalwijk, Rogier van der Sluijs, Roderick M. Houwert, Koen W. W. Lansink, Michael J. R. Edwards, Risco van Vliet, Thijs F. Verhagen, Nicolette Diets-Veenendaal, Luke P. H. Leenen, Mark van Heijl

**Affiliations:** 1 Department of Surgery, University Medical Center Utrecht, Utrecht, the Netherlands; 2 Department of Surgery, Maastricht University Medical Center, Maastricht, the Netherlands; 3 Centre for Artificial Intelligence in Medicine & Imaging, Stanford University, Stanford, California; 4 Traumazorgnetwerk Midden-Nederland, Utrecht, the Netherlands; 5 Department of Surgery, Elisabeth-TweeSteden Ziekenhuis, Tilburg, the Netherlands; 6 Netwerk Acute Zorg Brabant, Tilburg, the Netherlands; 7 Department of Surgery, Radboud University Medical Center, Nijmegen, the Netherlands; 8 Acute Zorgregio Oost, Nijmegen, the Netherlands; 9 Regionale Ambulance Voorziening Brabant Midden-West-Noord, 's-Hertogenbosch, the Netherlands; 10 Regionale Ambulance Voorziening Utrecht, Bilthoven, the Netherlands; 11 Department of Surgery, Diakonessenhuis Utrecht/Zeist/Doorn, Utrecht, the Netherlands

## Abstract

**Importance:**

Adequate prehospital triage is pivotal to enable optimal care in inclusive trauma systems and reduce avoidable mortality, lifelong disabilities, and costs. A model has been developed to improve the prehospital allocation of patients with traumatic injuries and was incorporated in an application (app) to be implemented in prehospital practice.

**Objective:**

To evaluate the association between the implementation of a trauma triage (TT) intervention with an app and prehospital mistriage among adult trauma patients.

**Design, Setting, and Participants:**

This population-based, prospective quality improvement study was conducted in 3 of the 11 Dutch trauma regions (27.3%), with full coverage of the corresponding emergency medical services (EMS) regions participating in this study. Participants included adult patients (age ≥16 years) with traumatic injuries who were transported by ambulance between February 1, 2015, and October 31, 2019, from the scene of injury to any emergency department in the participating trauma regions. Data were analyzed between July 2020 and June 2021.

**Exposures:**

Implementation of the TT app and the awareness of need for adequate triage created by its implementation (ie, the TT intervention).

**Main Outcomes and Measures:**

The primary outcome was prehospital mistriage, evaluated in terms of undertriage and overtriage. Undertriage was defined as the proportion of patients with an Injury Severity Score (ISS) of 16 or greater who were initially transported to a lower-level trauma center (designated to treat patients who are mildly and moderately injured) and overtriage as the proportion of patients with an ISS of less than 16 who were initially transported to a higher-level trauma center (designated to treat patients who are severely injured).

**Results:**

A total of 80 738 patients were included (40 427 [50.1%] before and 40 311 [49.9%] after implementation of the intervention), with a median (IQR) age of 63.2 (40.0-79.7) years and 40 132 (49.7%) male patients. Undertriage decreased from 370 of 1163 patients (31.8%) to 267 of 995 patients (26.8%), while overtriage rates did not increase (8202 of 39 264 patients [20.9%] vs 8039 of 39 316 patients [20.4%]). The implementation of the intervention was associated with a statistically significantly reduced risk for undertriage (crude risk ratio [RR], 0.95; 95% CI, 0.92 to 0.99, *P* = .01; adjusted RR, 0.85; 95% CI, 0.76-0.95; *P* = .004), but the risk for overtriage was unchanged (crude RR, 1.00; 95% CI, 0.99-1.00; *P* = .13; adjusted RR, 1.01; 95% CI, 0.98-1.03; *P* = .49).

**Conclusions and Relevance:**

In this quality improvement study, implementation of the TT intervention was associated with improvements in rates of undertriage. Further research is needed to assess whether these findings are generalizable to other trauma systems.

## Key Points

**Question:**

Was the implementation of the trauma triage intervention associated with a change in prehospital triage among adult patients with traumatic injuries?

**Findings:**

In this quality improvement study including 80 738 adult patients with traumatic injuries, the implementation of a trauma triage intervention with an application was associated with a statistically significantly lower adjusted risk for undertriage; the adjusted risk for overtriage was unchanged after the intervention.

**Meaning:**

This quality improvement study found that implementation of the trauma triage intervention was associated with improved rates of undertriage.

## Teaser

This quality improvement study evaluates the association between the implementation of the trauma triage (TT) intervention app and prehospital mistriage among adult patients with trauma.

## Introduction

Adequate prehospital triage is imperative to provide optimal care in inclusive trauma systems. Undertriage (ie, transporting patients in need of specialized care to lower-level trauma centers) is associated with avertible mortality and morbidity,^1^ whereas overtriage (ie, transporting patients who are mildly or moderately injured to higher-level trauma centers) can result in an overutilization of scarce resources and increased costs.^2^ Reducing undertriage generally takes precedence over decreasing overtriage, and the Dutch Health Care Institute and American College of Surgeons Committee on Trauma (ACSCOT) recommend to attain maximum undertriage rates of 10% and 5%, respectively.^3,4^ No inclusive trauma system worldwide is currently able to adhere with these guidelines while preserving acceptable overtriage rates.^5^ The ACSCOT does suggest overtriage rates up to 35% may be acceptable,^4^ but no universal agreement exists on acceptable overtriage rates.

In inclusive trauma systems, prehospital triage is generally performed by emergency medical services (EMS) professionals. These professionals assess a patient’s need for specialized trauma care at the scene of injury and subsequently decide trauma center is the most appropriate destination, given additional factors, such as patient acuity and trauma center proximity. EMS professionals in the Netherlands are aided in their decision-making by the field triage criteria of the National Protocol of Ambulance Services,^6^ which are derived from the American Field Triage Decision Scheme.^4,7^ Like previously developed prehospital triage tools, both decision schemes provide limited support to EMS professionals, as they were found to be relatively insensitive for identifying patients who are severely injured.^8,9,10^

As these triage protocols and tools are static flowcharts, unable to detect subtle and interacting patterns of signs and symptoms, a multivariable model was previously developed to improve the prehospital allocation of trauma patients. The model was found to potentially decrease the undertriage rate to approximately 10% while maintaining an overtriage rate of 50% in external validation.^11^ This model was integrated into a mobile application (app), to be implemented in prehospital practice as part of the trauma triage (TT) intervention. To our knowledge, the implementation of such a digital decision-support tool in prehospital clinical practice has not been studied before. The aim of this study was to evaluate the association of the implementation of the TT intervention with prehospital mistriage among adult patients with traumatic injuries.

## Methods

### Study Design, Setting, and Participants

This population-based, prospective quality improvement study was conducted between February 1, 2015, and October 31, 2019. The study was registered at the Dutch Trial Registry (NL6486). The Medical Ethical Committee of the University Medical Centre Utrecht determined the study was not subject to the Medical Research Involving Human Subjects Act and was therefore exempt from review and informed consent. This study is reported following the Standards for Quality Improvement Reporting Excellence (SQUIRE) reporting guideline.^12^

Of 11 Dutch inclusive trauma regions, 3 (27.3%; Traumazorgnetwerk Midden-Nederland, Netwerk Acute Zorg Brabant, and Acute Zorgregio Oost) participated in this study, which comprise 21 trauma centers: 3 higher-level (ie, level 1) trauma centers and 18 lower-level (ie, level 2 or 3) trauma centers. Corresponding EMS regions (ie, Utrecht and Brabant Midden-West-Noord*)* that are solely served by the participating EMS (ie, full coverage) participated. These EMS transport approximately 160 000 patients from the scene of injury to a trauma center annually^13^ and serve rural, suburban, and urban regions, covering approximately 5000 km^2^ and housing a population of approximately 4 million people (eFigure 1 in Supplement 1).

**Figure 1.**
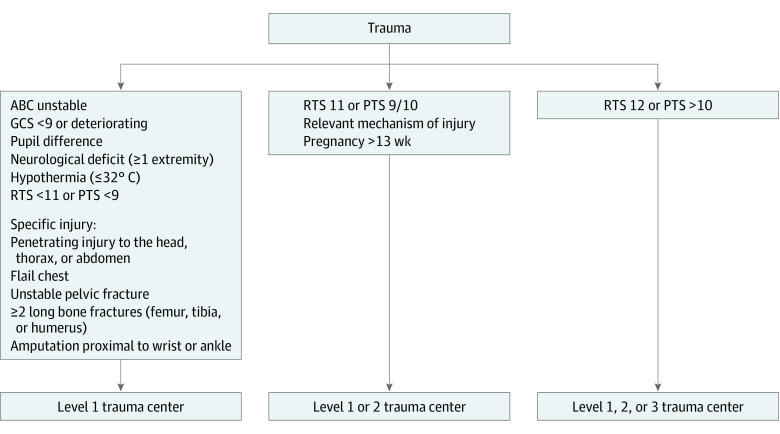
Field Triage Criteria of the Dutch National Protocol of Ambulance Services ABC indicates airway, breathing, or circulation; GCS, Glasgow Coma Scale; RTS, Revised Trauma Score; and PTS, Pediatric Trauma Score. In some areas with long expected transport times, it may be preferred to initially stabilize a patient with severe hemodynamic instability at the nearest hospital that is able to provide an adequate trauma response, if meeting a specialized physician (eg, trauma surgeon or anesthetist) during transport is not possible.

In the Netherlands, patients with traumatic injuries are generally transported to a hospital by ground ambulance and, in highly exceptional cases, by helicopter. Dutch ambulances are staffed with an EMS professional (licensed to provide prehospital advanced life support) and a dedicated driver (licensed to provide prehospital basic life support). Dutch EMS professionals are generally specialized nurses who previously worked as an emergency department nurse, intensive care unit nurse, or an anesthetic technician. In the Netherlands, a specialized physician (eg, trauma surgeon or anesthetist) is sent to the scene of injury to assist the EMS professional if the dispatch center expects a patient with seriously impaired vital functions. The Dutch National Protocol for Ambulance Services guides EMS professionals in their prehospital decision-making (Figure 1).^6^ The field triage criteria in this protocol for highest-level trauma care are comparable with those of the American Field Triage Decision Scheme.^4^ In the Netherlands, every inclusive trauma region has at least 1 higher-level (ie, level 1) trauma center. Such center meets the criteria needed to provide the highest level of trauma care, as outlined by the ACSCOT.^4,14^ Dutch level 2 and 3 facilities are considered lower-level trauma centers, designated to treat patients who are not severely injured.^14^ In the Netherlands, no patients with traumatic injuries are transported by ambulance to nontrauma centers.

Adult (age ≥16 years) patients with traumatic injuries who were transported during the inclusion period by a ground ambulance of a participating EMS from the scene of injury to any emergency department were included. Patients transported to a nonparticipating trauma region were excluded. Patients transported by EMS Utrecht were included between February 1, 2015, and September 30, 2019, and patients transported by EMS Brabant Midden-West-Noord were included between May 1, 2016, and October 31, 2019.

### TT Intervention

The TT intervention consisted of the implementation of the TT app and the awareness of the need for adequate triage created by its implementation. The TT app was used as a decision-support tool that, like the Dutch National Protocol of Ambulance Services, could be overruled by EMS professionals. As time is limited in prehospital triage, EMS professionals could generate recommendations without sending the result, could send it without filling out the patient record identifier, or could send the result with the patient record identifier. Results were sent to and saved at a secured server for medical data. The sample of patients in which the patient record identifier was registered could be linked to prehospital and hospital data to assess prehospital triage rates among these patients. The TT app was introduced to EMS professionals via email, a website,^15^ handouts, newsletters, posters, oral presentations at the EMS professionals’ team meetings, and an electronic learning seminar that was developed specifically for this study. These materials instructed on the importance of adequate prehospital triage, characteristics of undertriaged patients, current quality of triage by their EMS, and how to use the TT app. Various newsletters were sent to inform EMS professionals on the progress of the study, and multiple professionals from the participating EMS served as TT app ambassadors during the study.

**Figure 2.**
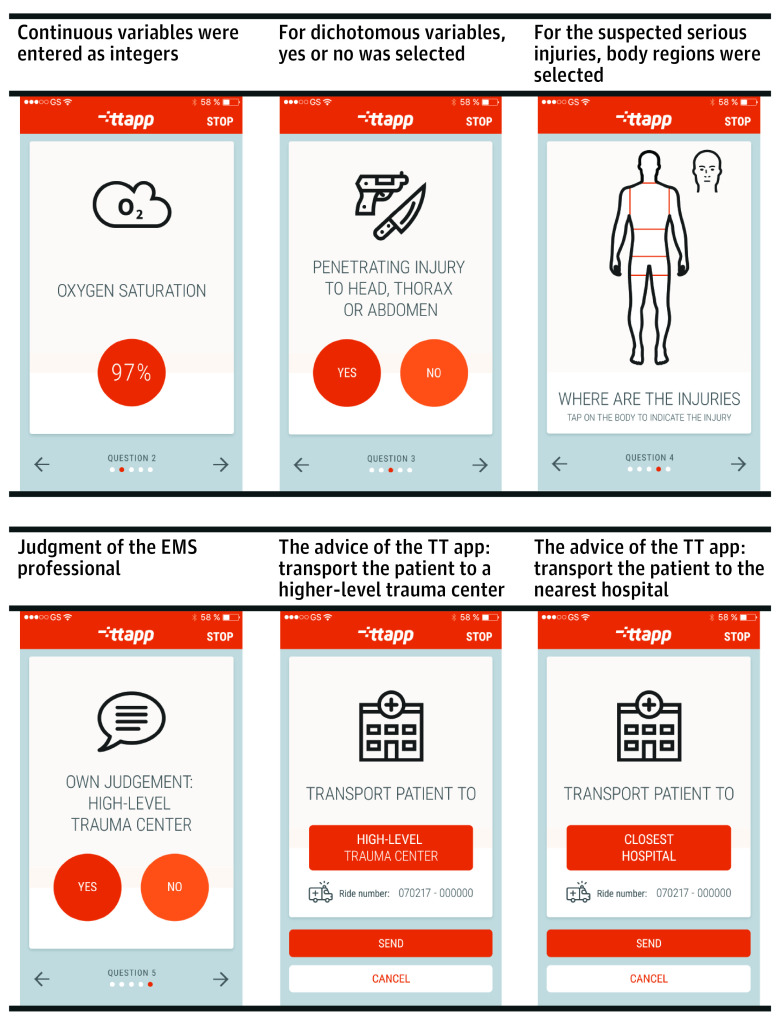
Usability of the Trauma Triage (TT) Application (App) EMS indicates Emergency Medical Services.

We hired a company specialized in constructing medical apps (Synappz Digital Healthcare) and developed the app in close collaboration with various EMS professionals. A model that included 10 variables (eFigure 2 and eAppendix in Supplement 1) was integrated in the app to provide recommendations. The model’s intercept was updated for EMS Brabant Midden-West-Noord to account for case-mix differences (eg, prevalence of severe injury).^11^ Probability thresholds for transport advice to a higher-level trauma center were chosen based on the maximum proportion of overtriage that was considered acceptable (ie, Utrecht, 50%; Brabant Midden-West-Noord, 35%) by the participating EMS and trauma regions. Age, oxygen saturation, and Glasgow Coma Scale score were entered as continuous variables, and EMS professionals selected either yes or no for the dichotomous variables mechanism criteria, burn wounds, and penetrating trauma (Figure 2). Regions suspected of serious injuries were selected on an illustration of a human body. After entering the model’s variables, the EMS professionals were asked their judgment on whether transport to a higher-level trauma center was indicated or not. Based on the 10 variables and the EMS professional’s judgment, the TT app provided a recommendation: transport to a higher-level trauma center or transport to the nearest trauma center. The TT app advised to transport a patient to a higher-level trauma center when the calculated probability was greater than the chosen threshold or when the EMS professional selected that higher-level trauma care was indicated. Implementation was preceded by a digital testing period to overcome technical problems. During the study, a mobile device was available in every ambulance to use the TT app: an iPhone 6s (Apple) in Utrecht and an iPad Air 2 (Apple) in Brabant Midden-West-Noord.

### Data Collection

Prehospital and hospital data were prospectively collected and linked with a previously validated accurate (accuracy, 100%; 95% CI, 100%-100%) linkage scheme.^16^ Further details regarding data collection are provided in the eMethods in Supplement 1.

### Outcomes

The primary outcome was prehospital mistriage, evaluated in terms of undertriage and overtriage. Undertriage was defined as the proportion of patients requiring specialized trauma care who were initially transported to a lower-level trauma center and overtriage as the proportion of patients not requiring specialized trauma care who were initially transported to a higher-level trauma center. Need for specialized trauma care was defined as an Injury Severity Score (ISS) of 16 or greater, which is the currently recommended reference standard to evaluate prehospital triage by the Dutch Health Care Institute and the ACSCOT.^4^ Secondary outcomes were mortality, quality of filling out variables in the TT app by EMS professionals, adherence with the advice of the TT app by EMS professionals, and prehospital triage in the sample of patients that was registered in the TT app.

### Statistical Analysis

The primary outcome was analyzed using before and after triage rates and crude and adjusted risk ratios (RRs) for both undertriage and overtriage. Crude and adjusted RRs were calculated using Poisson regression with robust SEs (ie, Zou modified Poisson regression).^17,18^ Confounders were chosen based on clinical reasoning and previous literature.^8,11,19,20,21^ Risks and odds were adjusted for age, sex, dispatch priority, day of the week, hour of the day, distance to the nearest higher-level trauma center, penetrating injury, hemodynamic instability (ie, compromised airway, breathing, or circulation), prehospital vital signs (systolic blood pressure, respiratory rate, Glasgow Coma Scale score, heart rate, and oxygen saturation), and ISS. Restricted cubic splines with 3 knots were used to account for nonlinearity. Sample size calculations were performed to estimate the required number of participants in our study to have sufficient power (ie, 80%) to show reduction of undertriage of 0.25 to 0.20 associated with implementation of the TT intervention with an app. This calculation showed that when using a statistical significance level of *P* = .05, the study needed to include 1092 patients who were severely injured before and 1092 patients who were severely injured after the intervention. As approximately 3% of the ambulance-transported patients with traumatic injuries were expected to be severely injured, we aimed to include at least 40 000 patients in the before group and 40 000 in the after group.

Mortality was analyzed using before and after mortality rates and crude and adjusted odds ratios (ORs) for death within 24 hours, within 48 hours, and during admission. Crude and adjusted ORs were calculated using generalized linear models with inverse probability weights. Weights were computed for all patients using the same potential confounders as for the primary analysis. Covariates were considered accurately balanced when a Pearson correlation of less than 0.1 was observed.^22^

The sample of patients registered in the TT app was used to analyze the other secondary outcomes. Quality of filling out variables in the TT app was analyzed by comparing the variables filled out by the EMS professionals with those coded by the researchers based on the free-text fields. Adherence was determined by comparing the advice provided by the TT app with the trauma center level of the transport destination. Quality of prehospital triage in these patients was analyzed using undertriage and overtriage rates.

Missing data were analyzed and assumed to be missing at random. Prehospital variables with missing values were age (0.03%), sex (0.02%), dispatch priority (0.007%), location of the scene of injury (0.1%), systolic blood pressure (15.2%), respiratory rate (19.5%), Glasgow Coma Scale score (7.0%), heart rate (12.7%), and oxygen saturation (20.0%). An estimation matrix was created to impute the missing values. The R packages mice and micemd (R Project for Statistical Computing) were used to perform multilevel multiple imputation to generate 30 different imputed data sets based on 20 iterations per set. All data sets were used to perform analyses of variables with missing data, which were subsequently pooled in accordance with the Rubin rules, if applicable. Sensitivity analyses were performed excluding the prehospital vital signs. All statistical analyses were performed using R version 4.0.3. *P* values were 2-sided, and statistical significance was set at *P* = .05. Data were analyzed from July 2020 and June 2021.

## Results

A total of 80 738 adult trauma patients were included, with a median (IQR) age of 63.2 (40.0-79.7) years and 40 132 (49.7%) were male patients; 40 427 patients (50.1%) were included before intervention implementation, and 40 311 patients (49.9%) were included after the implementation of the TT intervention (eFigure 3 in Supplement 1). No major differences were observed in patient demographic or prehospital characteristics (Table 1). Patients included after the intervention, compared with those included before the intervention, were less often severely injured (995 patients [2.5%] vs 1163 patients [2.9%]), admitted to the intensive care unit (1127 patients [2.8%] vs 1338 patients [3.3%]), and transported to a higher-level trauma center (8767 patients [21.7%] vs 8995 patients [22.2%]). Baseline characteristics of the individual EMS are provided in eTable 1 in Supplement 1.

**Table 1.** Baseline Characteristics of Patients Included Before and After Implementation of the Trauma Triage Intervention

Characteristic	No. (%)^a^	
	Before (n = 40 427)	After (n = 40 311)
Age, y		
Median (IQR)	62.6 (39.3-79.9)	63.8 (40.6-80.1)
>65	19 019 (47.0)	19 524 (48.5)
Sex		
Female	20 465 (50.6)	20 141 (49.9)
Male	19 962 (49.4)	20 170 (50.1)
Penetrating trauma	260 (0.6)	232 (0.6)
Assistance by a specialized physician	644 (1.6)	623 (1.5)
Prehospital vital signs		
Systolic blood pressure <90 mm Hg	487 (1.2)	520 (1.3)
Respiratory rate >29 or <10 per min	651 (1.6)	713 (1.8)
Glasgow Coma Scale score <13	1469 (3.6)	1450 (3.6)
Heart rate <40 or >100 beats per min	31 629 (78.2)	33 094 (82.1)
Oxygen saturation <90%	1403 (3.5)	1424 (3.5)
Severe injury (AIS ≥3)^b^		
Head	1106 (2.7)	1051 (2.6)
Face	35 (0.1)	52 (0.1)
Neck	24 (0.1)	18 (<0.1)
Thorax	898 (2.2)	896 (2.2)
Abdomen	152 (0.4)	127 (0.3)
Spine	263 (0.6)	258 (0.6)
Upper extremity	129 (0.3)	66 (0.2)
Lower extremity	4822 (11.9)	4579 (11.4)
Clinical characteristics		
Hospital admission	13 257 (32.8)	11 815 (29.3)
ICU admission	1338 (3.3)	1127 (2.8)
Prehospital triage		
ISS ≥16^c^	1163 (2.9)	995 (2.5)
Higher-level trauma center	8995 (22.2)	8767 (21.7)

Abbreviations: AIS, Abbreviated Injury Scale; ICU, intensive care unit; ISS, Injury Severity Score.

^a^ Age was missing in 0.03% of patients; sex, 0.02%, systolic blood pressure, 15.2%; respiratory rate, 19.5%;, Glasgow Coma Scale, 7.0%; heart rate, 12.7%; and oxygen saturation, 20.0%.

^b^ The AIS scoring system is used to classify injuries based on their severity (ranging from 1, indicating minor; to 6, unsurvivable) and body region (head or neck, face, chest, abdomen, extremity, external).

^c^ The ISS is the sum of the squares of the highest AIS code in the 3 most severely injured body regions and ranges from 0 to 75. An ISS of 16 or greater is generally considered major trauma that requires specialized or higher-level trauma care.

### Primary Outcome

The undertriage rate decreased from 370 of 1163 patients (31.8%) before the intervention to 267 of 995 patients (26.8%) after implementation of the intervention (crude RR, 0.95; 95% CI, 0.92-0.99; *P* = .01), while the overtriage rate did not increase (8202 of 39 264 patients [20.9%] vs 8039 of 39 316 patients [20.4%]; RR, 1.00; 95% CI, 0.99-1.00; *P* = .13) (Table 2). Implementation of the intervention was associated with statistically significantly lower adjusted risk for undertriage (RR, 0.85; 95% CI, 0.76-0.95; *P* = .004), but the adjusted risk for overtriage was unchanged (RR, 1.01; 95% CI, 0.98-1.03; *P* = .49). Undertriage decreased less in patients transported by EMS Brabant Midden-West-Noord than in patients transported by EMS Utrecht in both the crude (RR, 0.97; 95% CI, 0.93-1.03; *P* = .32; vs RR, 0.92; 95% CI, 0.87-0.98; *P* = .006) and adjusted (RR, 0.90; 95% CI, 0.78-1.03; *P* = .14; vs RR, 0.83; 95% CI, 0.70-0.98; *P* = .03) analyses (eTable 2 in Supplement 1).

**Table 2.** Prehospital Triage and Mortality Before and After Implementation of the Trauma Triage Intervention

Outcome	No. (%)		Crude analysis		Adjusted analysis		Sensitivity analysis^a^	
	Before	After	Estimate (95% CI)	*P* value	Estimate (95% CI)	*P* value	Estimate (95% CI)	*P* value
**Prehospital triage**								
Patient ISS, No.								
≥16	1163	995	NA	NA	NA	NA	NA	NA
<16	39 264	39 316	NA	NA	NA	NA	NA	NA
Undertriage^b^	370 (31.8)	267 (26.8)	0.95 (0.92-0.99)^c^	.01	0.85 (0.76-0.95)^d^	.004	0.84 (0.75-0.94)^d^	.002
Overtriage^e^	8202 (20.9)	8039 (20.4)	1.00 (0.99-1.00)^c^	.13	1.01 (0.98-1.03)^d^	.49	1.01 (0.99-1.04)^d^	.28
**Mortality**								
<24-h	94 (0.2)	65 (0.2)	0.69 (0.50-0.95)^f^	.02	0.71 (0.52-0.98)^g^	.04	0.71 (0.52-0.98)^g^	.04
<48-h	141 (0.4)	101 (0.3)	0.72 (0.56-0.93)^f^	.01	0.74 (0.58-0.96)^g^	.02	0.74 (0.74-0.96)^g^	.02
In-hospital	484 (1.2)	437 (1.1)	0.90 (0.79-1.03)^f^	.13	0.96 (0.84-1.09)^g^	.49	0.95 (0.83-1.08)^g^	.41

Abbreviations: ISS, Injury Severity Score; NA, not applicable.

^a^ Sensitivity analyses were calculated without prehospital vital signs.

^b^ Undertriage was defined as patients with ISS of 16 or greater who were transported to a lower-level (ie, level 2 or 3) trauma center.

^c^ Expressed as risk ratio, calculated using Poisson regression with robust standard errors (ie, Zou modified Poisson regression).

^d^ Expressed as adjusted risk ratio and adjusted for age, sex, dispatch priority, day of the week, hour of the day, distance to the nearest higher-level trauma center, penetrating injury, hemodynamic instability (comprised in airway, breathing, or circulation), pre-hospital vital signs (systolic blood pressure, respiratory rate, Glasgow Coma Scale, heart rate, and oxygen saturation), and ISS.

^e^ Overtriage was defined as patients with ISS less than 16 transported to a higher-level (ie, level 1) trauma center.

^f^ Expressed as odds ratio, calculated using generalized linear models with inverse probability weights.

^g^ Expressed as adjusted odds ratio and adjusted for age, sex, dispatch priority, day of the week, hour of the day, distance to the nearest higher-level trauma center, penetrating injury, hemodynamic instability (comprised in airway, breathing, or circulation), pre-hospital vital signs (systolic blood pressure, respiratory rate, Glasgow Coma Scale, heart rate, and oxygen saturation), and ISS.

### Secondary Outcomes

After implementation of the intervention, there were significant decreases in risks of death within 24 (adjusted OR, 0.71; 95% CI, 0.52-0.98; *P* = .04) or 48 (adjusted OR, 0.74; 95% CI, 0.58-0.96; *P* = .02) hours. The odds of in-hospital death did not change (adjusted OR, 0.96; 95% CI, 0.84-1.09; *P* = .49).

EMS professionals chose to send the result of the TT app to the secured server in 978 patients and registered the patient record identifier in 597 patients (61.0%). Details regarding use of the TT app, adherence with its advice, and prehospital triage in this sample of patients are displayed in Table 3. In 678 patients (69.3%), the app advised to transport a patient to a higher-level trauma center; 647 patients (66.2%) were registered as having been recommended by the model to be transported to a higher-level hospital, and 304 patients (31.1%) were noted by the EMS professional as needing to be transported to a higher-level hospital. Comparable results were found in the 597 patients registered with a patient record identifier (Table 3). The EMS records of these registrations were used by the researchers to manually code the variables of the model. When using these manually coded variables, the model would have recommended transport to a higher-level trauma center less often than the TT app recommended at the scene of injury (348 patients [58.3%] vs 415 patients [69.5%]). The differences between the registrations filled out by the EMS professional and researchers are displayed in eTable 3 in Supplement 1. The EMS professionals adhered to the advice of the TT app to transport a patient to a higher-level trauma center in 219 patients (50.2%), of whom 51 patients (23.3%) were severely injured. Nine patients (4.1%) for whom the EMS professional did not adhere to this advice were severely injured. None of the patients for whom the TT app advised transport to the nearest trauma center (rather than a higher-level center) was severely injured. The patients registered with patient record identifier were often severely injured (60 patients [10.1%]) and transported to a higher-level trauma center (253 patients [42.4%]). Among patients with patient record identifiers registered in the app, 9 patients (15.0%; 95% CI, 7.9%-26.3%) were undertriaged and 202 patients (37.6%; 95% CI, 33.6%-41.8%) were overtriaged.

**Table 3.** Registrations in the TT App Sent to the Secured Server by EMS Professionals

Measure	Registrations, No. (%)	
	Registration in TT app (n = 978)^a^	Registration in TT app with patient record identifier (n = 597)^a^
**Recommendation**		
App recommended higher-level trauma center^b^	678 (69.3)	436 (73.0)
Model recommended higher-level trauma center	647 (66.2)	415 (69.5)
Model would have recommended higher-level trauma center based on variables coded by the researchers	NA	348 (58.3)
EMS professional judged higher-level trauma center	304 (31.1)	191 (32.5)
**Adherence**		
TT app recommended transport to higher-level trauma center		
Patient transported to higher-level trauma center (adherent)	NA	219 (50.2)
Patients with ISS ≥16	NA	51 (23.3)
Patient transported to lower-level trauma center (nonadherent)	NA	217 (36.3)
Patients with ISS ≥16	NA	9 (4.1)
TT app recommended transport to lower-level trauma center		
Patient transported to higher-level trauma center (nonadherent)	NA	34 (5.7)
Patients with ISS ≥16	NA	0
Patient transported to lower-level trauma center (adherent)	NA	127 (5.7)
Patients with ISS ≥16	NA	0
**Prehospital triage**		
Patients with ISS ≥16	NA	60 (10.1)
Patients transported to higher-level trauma center	NA	253 (42.4)
Patients undertriaged, % (95% CI)	NA	15.0 (7.9-26.3)
Patients overtriaged, % (95% CI)	NA	37.6 (33.6-41.8)

Abbreviations: EMS, Emergency Medical Services; ISS, Injury Severity Score; NA, not applicable; TT app, trauma triage app.

^a^ As time is limited in prehospital triage EMS professionals could generate recommendations without sending the result, could send it without filling out the patient record identifier, or could send the result with the patient record identifier. The sample of patients in whom the patient record identifier was registered was linked to prehospital and hospital data to assess prehospital triage rates in this sample of patients.

^b^ The TT app advised transport to a higher-level trauma center when the calculated probability was greater than the chosen threshold or when the EMS professional selected that higher-level trauma care was indicated.

The registered cases of the individual EMS are provided in eTable 4 in Supplement 1. Patients registered with patient record identifiers who were transported by EMS Utrecht, compared with those transported by EMS Brabant Midden-West-Noord, were less often undertriaged (8.7%; 95% CI, 1.2%-28.0%; vs 18.9%; 95% CI, 9.2%-34.5%) and more often overtriaged (56.5%; 95% CI, 48.6%-64.1%; vs 30.0%; 95% CI, 25.6%-34.8%). The professionals of the EMS Brabant Midden-West-Noord less often adhered to the TT app recommending a higher-level hospital than the professionals of the EMS Utrecht (120 events [28.6%] vs 99 events [55.9%]).

## Discussion

This population-based quality improvement study investigated the association between the implementation of the TT intervention and prehospital mistriage. Patients transported after the implementation of the TT intervention had 15% less risk of being undertriaged, while their risk of being overtriaged did not change. Also, 24- and 48-hour mortality significantly decreased after implementation of the app, while in-hospital death did not change. A reduction of undertriage was observed in EMS region Utrecht, while there was no statistically significant difference in EMS region Brabant Midden-West-Noord, presumably due to the relatively higher chosen probability threshold, lower adherence to the advice provided by the TT-app, and longer distances in Brabant Midden-West-Noord.

Prior studies found that currently used field triage protocols and previously developed triage tools are unable to adequately identify patients in need of higher-level trauma care.^8,9,10^ Their criteria are too generic, since trauma is caused by a wide variety of mechanisms and patients in need of higher-level trauma care can present in a multitude of ways. Moreover, important information is lost by the fact that criteria of the currently used protocols do not interact and continuous criteria are often dichotomized (eg, systolic blood pressure <90 mm Hg).^4,6^ In contrast to these protocols, a model is able to produce advice on an individual patient level and could be improved by providing additional data (eg, machine learning). Since the introduction of the first triage tools, there is an ongoing debate regarding the relationship between such tools and the judgment of EMS professionals.^23^ Some found the judgment of EMS professionals to be insensitive,^24^ accurate,^25^ or superior^26^ compared with triage tools. Others have suggested combining such rules with the judgment of EMS professionals to improve prehospital triage.^27,28^ In our opinion, providing EMS professionals with advice based on personalized estimations is an elegant method to combine previously gathered knowledge with the judgment of EMS professionals. Moreover, the use of a model incorporated in a mobile device enables EMS and trauma regions to continuously adapt prehospital triage based on local needs and agreements (eg, adjust probability thresholds based on acceptable overtriage rates).

This study found that the TT intervention could successfully be implemented in approximately 25% of the Dutch trauma system. Its implementation was significantly associated with a lower risk for undertriage, while nationwide such trend was not observed.^29^ Also, the intervention was associated with improved chances of survival for patients, as we found a significant decrease of death within the first days of admission. Further research in which interhospital transfers are taken into account, as these are known to affect chances of survival,^30^ is needed to determine definite triage rates and draw conclusions regarding the association of the intervention with mortality.

To further improve prehospital triage, we recommend making the TT app available to be used in all Dutch prehospital trauma patients, especially in those where doubt exists regarding a patient’s need for higher-level trauma care. In this study, the first version of the TT intervention was investigated. Further research is needed to assess the effect of updating the TT app and incorporated model on prehospital triage. A next step would be to investigate the impact of the updated intervention in the form a stepped-wedge cluster-randomized trial. Also, additional research is needed to assess whether our findings are generalizable to other trauma systems. To aid in assessing this, the studied model will be available for other researchers.

Strengths of this study include its extensive implementation strategy, its selection of participants, and standardized data collection. First, the researchers and participating EMS used an extensive strategy to instruct as many EMS professionals as possible on the importance of accurate prehospital triage and how to adequately use the TT app. Second, patients were not selected solely on chief concern (eg, trauma), but were selected by using a highly accurate and previously validated selection tool.^16^ As ambulance-transported patients often have multiple concerns, the use of such a selection tool is pivotal to minimize the chance of introducing selection bias. Moreover, selection bias was minimized by the fact that the participating regions were solely served by the participating EMS (ie, full coverage). Furthermore, approximately 25% of Dutch trauma centers participated in this study, and the participating EMS serve different types of areas (ie, urban, suburban, and rural),^31^ which increases the generalizability of our results. Third, patient records were prospectively and consistently collected by the participating EMSs and trauma regions. Moreover, EMS records of the patients that could be linked to registrations in the TT app were assessed by the researchers in a standardized manner and blinded from the hospital outcomes, and all hospital data were coded by the data registrars according to the standards of the registry without access to the EMS records. Furthermore, the Dutch Trauma Registry includes all patients with traumatic injuries (regardless of a patient’s age or injury severity) who were admitted to any trauma center (ie, any trauma-receiving hospital).^32^ Additionally, a deterministic and probabilistic linking scheme that was previously validated showing an high accuracy was used to link the EMS records to the hospital outcome data.^16^

### Limitations

This study has several limitations. First, EMS professionals chose to send the result of the TT app to the secured server in a minority of patients. According to feedback received from the EMS professionals, they regularly forgot to send the result after using the app, which should be resolved in next a version or phase by, for example, integrating it in the digital EMS record. This could also overcome the unnecessary duplicate variable registration (ie, in the application and EMS record) EMS professionals encountered. Moreover, as this was the first study that tested the model and app in prehospital clinical practice, EMS professionals could not be obligated to use the app or send the result to the secured server. They used the application particularly when they were in doubt regarding a patient’s need for higher-level trauma care (ie, not for patients who were clearly mildly or severely injured). Increased or standard use of the TT app could potentially lead to further improvement of prehospital triage. Second, although the app was developed in close collaboration with various EMS professionals and strict instructions were provided regarding its use, EMS professionals filled out the prehospital factors differently than the researchers. This was mainly related to the fact that EMS professionals in certain cases also selected body injuries if only a minor injury was suspected, which likely resulted too often in a recommendation to transport a patient to a higher-level trauma center. Third, the model integrated in the TT app was developed based on a relatively small population (4950 patients, including 435 patients who were severely injured) that was selected on chief concern (ie, trauma). Moreover, the model was developed only to identify patients who were severely injured as ISS of 16 or greater, which may not be the best method, as ISS of 16 or greater does not thoroughly correlate with a patient’s need for critical-resources.^33,34^ In future studies, the model should be updated to improve its performance and generalizability across different EMS using a consensus-based dependent variable consisting of, among others, critical resources.^35,36^

## Conclusions

In this quality improvement study, implementation of the TT app intervention was associated with lower rates of prehospital undertriage. Supporting EMS professionals in their decision-making by calculating an individual patient’s probability to be in need of specialized care at the scene of injury is a novel and promising approach to optimize field triage.

## Notes

Supplement 1. **eTable 1.** Baseline Characteristics of the Participants Included Before and After Implementation of the Trauma Triage Intervention, Stratified by Individual Emergency Medical Services **eTable 2.** Prehospital Triage Before and After Implementation of the Trauma Triage Intervention by the Individual Emergency Medical Services **eTable 3.** Differences in Registration of Variables Between EMS Professionals and Researchers **eTable 4.** Registrations in the TT App Sent to the Secured Server by EMS Professionals by Individual Emergency Medical Services **eFigure 1.** Participating Regions **eFigure 2.** Model Integrated in the Trauma Triage App **eFigure 3.** Flowchart of Patient Enrollment
eMethods.
**eAppendix.** R Script of the Algorithm

Supplement 2.
Data Sharing Statement
