# Supplementary material for: Accuracy of Prehospital Triage of Adult Patients With Traumatic Injuries Following Implementation of a Trauma Triage Intervention
Source: JAMA Netw Open. 2023 Apr 4;6(4):e236805. doi: 10.1001/jamanetworkopen.2023.6805 (PMC10074221; doi:10.1001/jamanetworkopen.2023.6805)
Supplement: Supplement 1. — eTable 1. Baseline Characteristics of the Participants Included Before and After Implementation of the Trauma Triage Intervention, Stratified by Individual Emergency Medical Services eTable 2. Prehospital Triage Before and After Implementation of the Trauma Triage Intervention by the Individual Emergency Medical Services eTable 3. Differences in Registration of Variables Between EMS Professionals and Researchers eTable 4. Registrations in the TT App Sent to the Secured Server by EMS Professionals by Individual Emergency Medical Services eFigure 1. Participating Regions eFigure 2. Model Integrated in the Trauma Triage App eFigure 3. Flowchart of Patient Enrollment eMethods. eAppendix. R Script of the Algorithm [file jamanetwopen-e236805-s001.pdf]

Supplemental Online Content

Lokerman RD, van Rein EJ, Waalwijk JF, et al; Prehospital Trauma Triage Research Collaborative (PTTRC). Accuracy of prehospital triage of adult patients with traumatic injuries following implementation of a trauma triage intervention. *JAMA Netw Open*. 2023;6(4):e236805. doi:10.1001/jamanetworkopen.2023.6805

eTable 1. Baseline Characteristics of the Participants Included Before and After Implementation of the Trauma Triage Intervention, Stratified by Individual Emergency Medical Services
eTable 2. Prehospital Triage Before and After Implementation of the Trauma Triage Intervention by the Individual Emergency Medical Services
eTable 3. Differences in Registration of Variables Between EMS Professionals and Researchers
eTable 4. Registrations in the TT App Sent to the Secured Server by EMS Professionals by Individual Emergency Medical Services
eFigure 1. Participating Regions
eFigure 2. Model Integrated in the Trauma Triage App
eFigure 3. Flowchart of Patient Enrollment
eMethods.
eAppendix. R Script of the Algorithm

This supplemental material has been provided by the authors to give readers additional information about their work.

© 2023 Lokerman RD et al. *JAMA Netw Open.*

eTable 1. Baseline Characteristics of the Participants Included Before and After Implementation of the Trauma Triage Intervention, Stratified by Individual Emergency Medical Services

Characteristic	**EMS Utrecht before** (n = 14 895)	**EMS Utrecht after** (n = 14 842)	**EMS Brabant Midden-West-Noord before** (n = 25 532)	**EMS Brabant Midden-West-Noord after** (n = 25 469)
Age, median (IQR), y	61.3 (36.9–79.8)	63.0 (39.1–80.3)	63.3 (40.6–79.9)	64.3 (41.4–79.9)
Age ≥65 years	6778 (45.5)	7024 (47.3)	12 241 (47.9)	12 500 (49.1)
Sex, No. (%)				
Female	7809 (52.4)	7759 (52.3)	12 656 (49.6)	12 382 (48.6)
Male	7086 (47.6)	7083 (47.7)	12 876 (50.4)	13 087 (51.4)
Penetrating trauma, No. (%)	118 (0.8)	85 (0.6)	142 (0.6)	147 (0.6)
Assistance by a specialized physician, No. (%)	111 (0.7)	114 (0.8)	553 (2.2)	509 (2.0)
Pre-hospital vital signs, No. (%)				
Systolic blood pressure <90 mmHg	189 (1.3)	221 (1.5)	298 (1.2)	299 (1.2)
Respiratory rate >29 or <10 per min	270 (1.8)	282 (1.9)	381 (1.5)	431 (1.7)
Glasgow Coma Scale <13	579 (3.9)	597 (4.0)	890 (3.5)	853 (3.3)
Heart rate <40 or >100 per min	13 039 (87.5)	13 283 (89.5)	18 590 (72.8)	19 811 (77.8)
Oxygen saturation <90%	775 (5.2)	795 (5.4)	628 (2.5)	629 (2.5)
Severe injury (AIS[a] score ≥3), No. (%)				
Head	491 (3.3)	459 (3.1)	615 (2.4)	592 (2.3)
Face	6 (<0.1)	11 (0.1)	29 (0.1)	41 (0.2)
Neck	12 (0.1)	11 (0.1)	12 (<0.1)	7 (<0.1)
Thorax	405 (2.7)	437 (2.9)	493 (1.9)	459 (1.8)
Abdomen	59 (0.4)	59 (0.4)	93 (0.4)	68 (0.3)
Spine	117 (0.8)	109 (0.7)	146 (0.6)	149 (0.6)
Upper extremity	96 (0.6)	32 (0.2)	33 (0.1)	34 (0.1)
Lower extremity	1923 (12.9)	1859 (12.5)	2899 (11.4)	2720 (10.7)
Clinical characteristics, No. (%)				
Hospital admission	5202 (34.9)	4977 (33.5)	8055 (31.5)	6838 (26.8)
ICU admission	581 (3.9)	526 (3.5)	484 (1.9)	397 (1.6)
Pre-hospital triage, No. (%)				
ISS[b] ≥16	488 (3.2)	432 (2.9)	675 (2.6)	563 (2.2)
Higher-level trauma center	2323 (15.6)	2187 (14.7)	6672 (26.1)	6580 (25.8)

Abbreviations: AIS, Abbreviated Injury Scale; EMS, Emergency Medical Service; ICU, Intensive Care Unit; ISS, Injury Severity Score. Age missed in 0.03%, gender in 0.02%, systolic blood pressure missed in 15.2%, respiratory rate in 19.5%, Glasgow Coma Scale in 7.0%, heart rate in 12.7%, and oxygen saturation in 20.0% of the patients.

[a] The Abbreviated Injury Scale (AIS) scoring system is used to classify injuries based on their severity (ranging from 1/minor to 6/unsurvivable) and body region (head/neck, face, chest, abdomen, extremity, external).

[b] The Injury Severity Score (ISS) is the sum of the squares of the highest AIS code in the three most severely injured body regions and ranges from 0 to 75. An ISS of 16 or greater is generally considered major trauma, which requires specialized/higher-level trauma care.

eTable 2. Prehospital Triage Before and After Implementation of the Trauma Triage Intervention by the Individual Emergency Medical Services

Pre-hospital triage			Before	After	Crude analysis: Risk ratio (95% CI)	Crude analysis: P-value	Adjusted analysis[a]: Risk ratio (95% CI)	Adjusted analysis[a]: P-value	Sensitivity analysis[c]: Risk ratio (95% CI)	Sensitivity analysis[c]: P-value
EMS Utrecht	Undertriage	No.	163	109	0.92 (0.87-0.98)	.006	0.82 (0.69-0.98)	.03	0.82 (0.68-0.98)	0.03
		ISS ≥16, No.	488	432						
		Rate, %	33.4	25.2						
	Overtriage	No.	1998	1864	0.99 (0.98-1.00)	.02	0.96 (0.91-1.02)	.16	0.97 (0.92-1.02)	0.27
		ISS ≥16, No.	14 407	14 410						
		Rate, %	13.9	12.9						
EMS Brabant Midden-West-Noord	Undertriage	No.	207	158	0.97 (0.93-1.03)	.32	0.90 (0.79-1.03)	.14	0.90 (0.78-1.03)	0.13
		ISS ≥16, No.	675	563						
		Rate, %	30.7	28.1%						
	Overtriage	No.	6204	6175	1.00 (0.99-1.01)	.67	1.03 (1.01-1.05)	.01	1.03 (1.01-1.05)	.005
		ISS ≥16, No.	25 325	25 311						
		Rate, %	25.0	24.8						

Mortality			Before	After	Crude analysis: Odds ratio (95% CI)	Crude analysis: P-value	Adjusted analysis[b]: Odds ratio (95% CI)	Adjusted analysis[b]: P-value	Sensitivity analysis[c]: Odds ratio (95% CI)	Sensitivity analysis[c]: P-value
EMS Utrecht	<24 hours	No.	49	27	0.55 (0.34-0.88)	0.01	0.59 (0.37-0.94)	0.03	0.58 (0.36-0.94)	0.03
		Rate, %	0.33	0.18						
	<48 hours	No.	76	42	0.55 (0.38-0.81)	0.002	0.57 (0.39-0.84)	0.004	0.57 (0.39-0.84)	0.004
		Rate, %	0.51	0.28						
	In-hospital	No.	209	175	0.84 (0.68-1.03)	0.09	0.85 (0.69-1.04)	0.11	0.86 (0.70-1.05)	0.14
		Rate, %	1.40	1.18						
EMS Brabant Midden-West-Noord	<24 hours	No.	45	38	0.85 (0.55-1.30)	0.45	0.86 (0.56-1.32)	0.49	0.84 (0.55-1.30)	0.44
		Rate, %	0.18	0.15						
	<48 hours	No.	65	59	0.91 (0.64-1.29)	0.60	0.94 (0.67-1.34)	0.75	0.91 (0.64-1.29)	0.60
		Rate, %	0.25	0.23						
	In-hospital	No.	275	262	0.95 (0.81-1.13)	0.59	1.03 (0.87-1.22)	0.71	1.01 (0.85-1.20)	0.88
		Rate, %	1.08	1.03						

© 2023 Lokerman RD et al. *JAMA Netw Open.*

Abbreviation: EMS, Emergency Medical Service.
[a] Crude and adjusted risk ratios were calculated using poisson regression with robust standard errors (ie, Zou's modified poisson regression).
[b] Adjusted odds ratios were calculated using generalized linear models with inverse probability weights.
Risks and odds were adjusted for age, gender, dispatch priority, day of the week, hour of the day, distance to the nearest higher-level trauma center, penetrating injury, hemodynamic instability (comprised in airway, breathing, or circulation), pre-hospital vital signs (systolic blood pressure, respiratory rate, Glasgow Coma Scale, heart rate, and oxygen saturation), and ISS.
[c] Sensitivity analyses without pre-hospital vital signs.
Age was missing in 0.03%, gender in 0.02%, dispatch priority in 0.007%, scene of injury location in 0.1%, systolic blood pressure in 15.2%, respiratory rate in 19.5%, Glasgow Coma Scale in 7.0%, heart rate in 12.7%, and oxygen saturation in 20.0% of the patients, all were multiply imputed.

© 2023 Lokerman RD et al. *JAMA Netw Open.*

eTable 3. Differences in Registration of Variables Between EMS Professionals and Researchers

Model predictors and outcome	TT-app used with registration of patient-record-identifier[a] (n = 597)	
	**Filled out EMS professional**	**Filled out by researchers**
**Predictors model TT-app**		
Age, median (IQR), y	55 (33–72)	55.2 (33.1–72.0)
Physiologic characteristics, No. (%)		
Oxygen saturation <90%	12 (2.0)	23 (3.9)
Glasgow Coma Scale <13	39 (6.5)	42 (7.0)
Mechanism of injury, No. (%)		
Mechanism criteria	168 (28.1)	72 (12.1)
Burns or smoke inhalation	7 (1.2)	4 (0.7)
Penetrating injury to the head, thorax, or Abdomen	9 (1.5)	4 (0.7)
Injury characteristics, No. (%)		
Suspected of a serious injury to the head, cervical spine, or neck	220 (36.9)	243 (40.7)
Suspected of a serious injury to the thorax	44 (7.4)	88 (14.7)
Suspected of a serious injury to the pelvis	137 (22.9)	20 (3.4)
Suspected of a serious injury in ≥2 body regions (head/neck, face, thorax, abdomen, pelvis, extremities)	208 (34.8)	143 (24.0)
Suspected of a serious injury to the face	154 (25.8)	59 (9.9)
Suspected of a serious injury to the abdomen	71 (11.9)	34 (5.7)
Suspected of a serious injury to the extremities	226 (37.9)	209 (35.0)
**Outcome model TT-app**		
Transport to a higher-level trauma center, No. (%)	415 (69.5)	348 (58.3)

Abbreviations: EMS, Emergency Medical Services; TT-app, trauma triage app.
Glasgow Coma Scale missed in 1.5% and oxygen saturation in 9.5% of the patients filled out by the researchers. All were multiply imputed and values derived from multiply imputed variables were rounded to zero decimals.
[a] As time is limited in pre-hospital triage EMS professionals could generate predictions without sending the result, could send it without filling out the patient-record-identifier, or could send the result with the patient-record-identifier. The sample of patients in whom the patient-record-identifier was registered, was linked to pre-hospital and hospital data to assess pre-hospital triage rates in this sample of patients.

© 2023 Lokerman RD et al. *JAMA Netw Open.*

eTable 4. Registrations in the TT App Sent to the Secured Server by EMS Professionals by Individual Emergency Medical Services

**Secondary outcomes**	**Registration in TT-app EMS Utrecht**[a] (n = 346)	**TT-app used with registration of patient-record-identifier EMS Utrecht**[a] (n = 177)		**Registration in TT-app EMS Brabant Midden-West-Noord**[a] (n = 632)	**TT-app used with registration of patient-record-identifier EMS Brabant Midden-West-Noord**[a] (n = 420)	
		**Filled out by EMS professional**	**Filled out by researchers**		**Filled out by EMS professional**	**Filled out by researchers**
Advice TT-app, No. (%)						
Transport to level 1 trauma center	245 (70.8)	130 (73.4)	-	433 (68.5)	306 (72.9)	-
Model +	232 (67.1)	112 (68.9)	102 (57.6)	415 (65.7)	293 (69.8)	246 (58.7)[b]
Judgment of EMS professional +	146 (42.2)	89 (50.3)	-	158 (25.0)	105 (25.0)	-
Compliance, No. (%)						
TT-app + & higher-level trauma center	-	99 (55.9)	-	-	120 (28.6)	-
IS ≥16	-	21 (21.2)	-	-	30 (25.0)	-
TT-app + & lower-level trauma center	-	31 (17.5)	-	-	186 (44.3)	-
ISS ≥16	-	2 (6.5)	-	-	7 (3.7)	-
TT-app - & higher-level trauma center	-	9 (5.1)	-	-	25 (5.9)	-
ISS ≥16	-	0	-	-	0	-
TT-app - & lower-level trauma center	-	38 (21.5)	-	-	89 (21.2)	-
ISS ≥16	-	0	-	-	0	-
Pre-hospital triage, No. (%)						
ISS ≥16	-	23 (13.0)		-	37 (8.8)	
Transport to higher-level trauma center	-	108 (61.0)		-	145 (34.5)	
Undertriage, % (95% CI)	-	8.7 (1.2–28.0)		-	18.9 (9.2–34.5)	
Overtriage, % (95% CI)	-	56.5 (48.6–64.1)		-	30.0 (25.6–34.8)	

Abbreviations: EMS, Emergency Medical Services; ISS, Injury Severity Score; TT-app, trauma triage app.
[a] As time is limited in pre-hospital triage EMS professionals could generate predictions without sending the result, could send it without filling out the patient-record-identifier, or could send the result with the patient-record-identifier. The sample of patients in whom the patient-record-identifier was registered, was linked to pre-hospital and hospital data to assess pre-hospital triage rates in this sample of patients.
[b] Glasgow Coma Scale missed in 1.5% and oxygen saturation in 9.5% of the patients filled out by the researchers, all were multiply imputed.

© 2023 Lokerman RD et al. *JAMA Netw Open.*

eFigure 1. Participating Regions

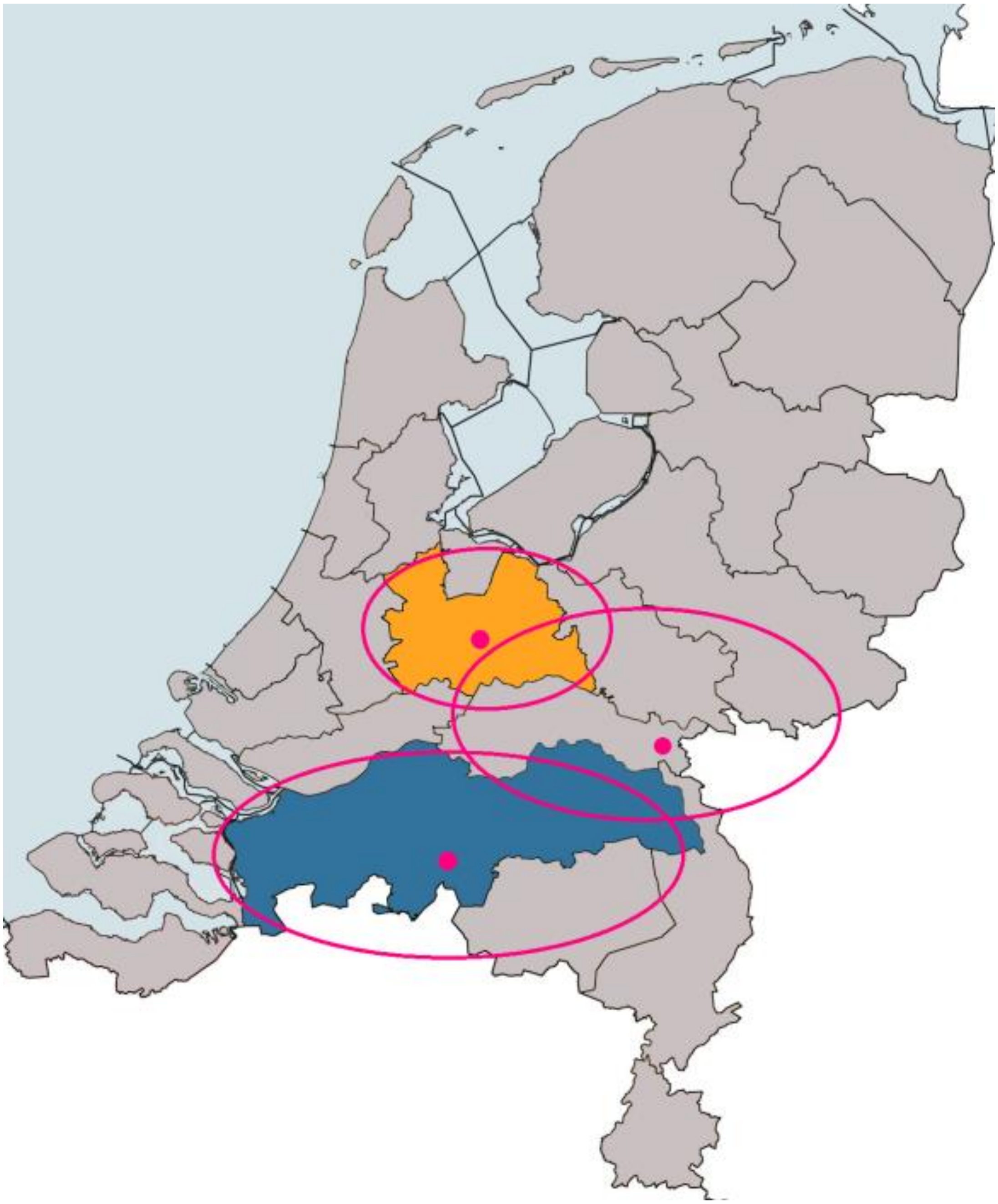

Yellow: Emergency Medical Services (EMS) region RAVU.

Blue: Emergency Medical Services (EMS) region Brabant Midden-West-Noord.

Purple: participating trauma regions (i.e., Traumazorgnetwerk Midden-Nederland, Acute Zorgregio Oost, and Netwerk Acute Zorg Brabant) and higher-level trauma centers.

© 2023 Lokerman RD et al. *JAMA Netw Open.*

eFigure 2. Model Integrated in the Trauma Triage App

Intercept EMS *Utrecht*	Intercept EMS *Brabant Midden-West-Noord*
6.064	5.322

Age
+ 0.013

Oxygen Saturation
− 0.062

Glasgow Coma Scale
− 0.314

Mechanism criteria
+ 1.310

Burns/smoke inhalation
+ 1.914

Penetrating injury
+ 1.245

Serious injury head/neck
+ 0.943

Serious injury thorax
+ 0.456

Serious injury pelvis
+ 2.776

Serious injury ≥2 regions
+ 0.713

Thresholds for transport to a higher-level trauma centre: Emergency Medical Services (EMS) Utrecht 0.03774761 and EMS Brabant Midden-West-Noord 0.0145000. The mechanism criteria were considered present if the patient fell from ≥2 meters, suffered from a motorcycle accident ≥32 km/h, or was entrapped in a motorised vehicle. Burns were considered present if a second-degree burn was suspected. A serious injury was present in case an injury was suspected that corresponded with an Abbreviated Injury Scale score ≥2. The following six body regions were considered independent regions for the variable serious injury ≥2 regions: head/neck, face, thorax, abdomen, pelvis, and extremities.

© 2023 Lokerman RD et al. *JAMA Netw Open.*

eFigure 3. Flowchart of Patient Enrollment

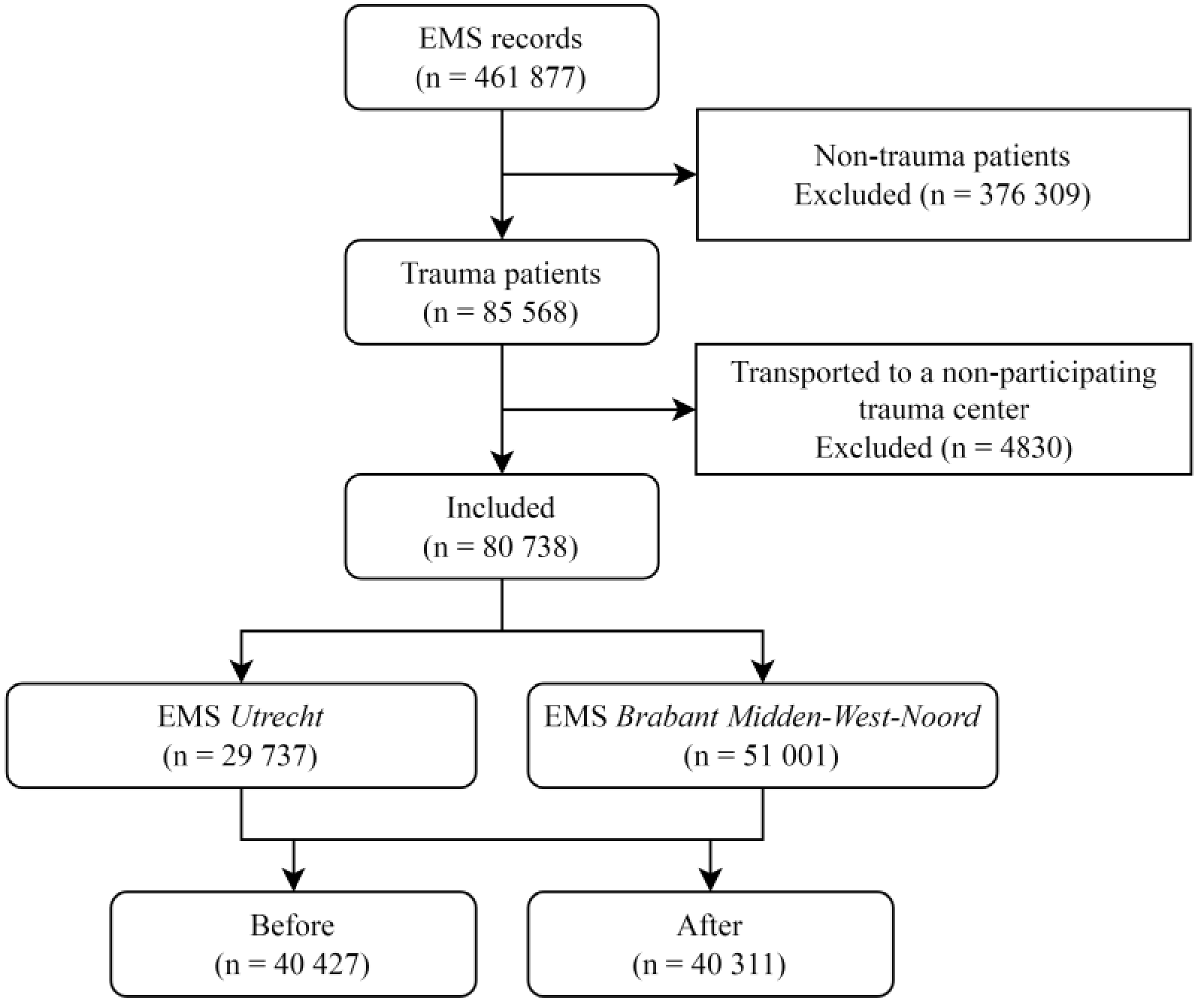

Abbreviation: EMS, Emergency Medical Services.

© 2023 Lokerman RD et al. *JAMA Netw Open.*

eMethods.

Data collection

Electronic EMS records were prospectively collected and comprised, among others, patient characteristics, on-scene vital signs, and free-text fields that were filled out by EMS professionals. The latter contain, among others, a description of the trauma mechanism, diagnostic findings, and considerations regarding provided prehospital treatments. The prehospital variables and free-text fields were used to select the records of trauma patients with an previously developed and externally validated prediction model, with an externally validated accuracy of 98.9% (95% CI, 98.3–99.2).[1] The EMS records were linked to data collected for the Dutch Trauma Registry by the three participating trauma regions. This registry prospectively includes all admitted trauma patients[2] and electronically documents, among others, all injuries diagnosed within the first 30 days post-trauma and various clinical outcomes (eg, mortality). Trained data registrars collected these data, classified injuries using the Abbreviated Injury Scale 2005, update 2008, and computed Injury Severity Scores (ISS). Combined deterministic and probabilistic linkage was applied to link the EMS records to the data of the registry. The patient-record-identifier was used to perform deterministic linkage and probabilistic linkage was conducted with a prediction model, which comprised multiple characteristics of the patient/accident (eg, date of injury). This linking method previously was shown to have an accuracy of 100.0% (95% CI, 100.0–100.0).[1] The EMS records of the patients which could be linked to registrations in the TT-app were assessed by the researchers, in a standardized manner and blinded from the hospital outcomes, to code the prehospital predictors of the model included in the TT-app. The hospital data were coded by the data registrars according to the standards of the registry without access to the EMS records.

© 2023 Lokerman RD et al. *JAMA Netw Open.*

Zip codes of the scene of injury and exact addresses of hospitals were converted into latitude and longitude coordinates with Open Street Map (OpenStreetMap, Cambridge, UK).[3] Previous research showed that actual driving distances could reliably be estimated using zip codes.[4] The two nearest higher-level trauma centers were selected based on the haversine method and subsequently we computed the transport times to both centers using Bing Maps (Microsoft™, Redmond, US).[5] The shortest driving distance was selected as the estimated driving distance from the scene to the nearest higher-level trauma center.

References

1. van der Sluijs R, Lokerman RD, Waalwijk JF, et al. Accuracy of pre-hospital trauma 534 triage and field triage decision rules in children (P2-T2 study): an observational study. 535 Lancet Child Adolesc Health 2020.
2. van der Vliet QMJ, Hietbrink F, Leenen LPH. Inclusion of All Patients Admitted for 537 Trauma in Trauma Registries. JAMA Surg 2019.
3. Zorginstituut Nederland. Spoed moet goed: indicatoren en normen voor zes spoedzorgindicaties. 2015.
4. Jarman MP, Sturgeon D, Mathews I, et al. Validation of Zip Code-Based Estimates of Ambulance Driving Distance to Control for Access to Care in Emergency Surgery Research. JAMA Surg 2019; 154(10):970-971.
5. Bing Maps. Microsoft Cooperation. Available at: https://www.bing.com/maps. Published 2020. Accessed 05-02-2020.

© 2023 Lokerman RD et al. *JAMA Netw Open.*

R-script of the algorithm

```
# Install packages
install.packages("tidyverse")
library(tidyverse)

# Import the predictors in dataframe A from Rds or CVS

# As RDS
A <- readRDS("H:\\Data/Name_Dataset.Rds")

# As CVS
A <- read_csv2(
  file = "H:/Data/Name_Dataset.csv",
  na = c("?", "NA", "", " ")
)

### Utrecht ###

# Import model
Model <- function(
  Age = NA,
  SPO2 = NA,
  GCS = NA,
  Burns_Inhalation = NA,
  Mechanism_Criteria = NA,
  Penetrating = NA,
  Head_Neck = NA,
  Thorax = NA,
  Pelvic = NA,
  Multiple_Regions = NA
) {
  6.064 +
  0.013*Age -
  0.062*SPO2 -
  0.314*GCS +
  1.914*(Burns_Inhalation == TRUE) +
  1.310*(Mechanism_Criteria == TRUE) +
  1.245*(Penetrating == TRUE) +
  0.943*(Head_Neck == TRUE) +
  0.456*(Thorax == TRUE) +
  2.776*(Pelvic == TRUE) +
  0.713*(Multiple_Regions == TRUE)
}
```

© 2023 Lokerman RD et al. *JAMA Netw Open.*

```
# Calculate linear predictors by hand
A$Linear_Predictor <- Model(
  A$Age,
  A$SPO2,
  A$GCS,
  A$Burns_Inhalation,
  A$Mechanism_Criteria,
  A$Penetrating,
  A$Head_Neck,
  A$Thorax,
  A$Pelvic,
  A$Multiple_Regions
)

# Calculate probabilities
A$Probabilities <- (1/(1+exp(-A$Linear_Predictor)))

# Calculate predictions with threshold
A$Predictions <- ifelse(A$Probabilities > 0.03901, 1, 0)

### Brabant Midden-West-Noord ###

# Import model
Model <- function(
  Age = NA,
  SPO2 = NA,
  GCS = NA,
  Burns_Inhalation = NA,
  Mechanism_Criteria = NA,
  Penetrating = NA,
  Head_Neck = NA,
  Thorax = NA,
  Pelvic = NA,
  Multiple_Regions = NA
) {
  5.322 +
    0.013*Age -
    0.062*SPO2 -
    0.314*GCS +
    1.914*(Burns_Inhalation == TRUE) +
    1.310*(Mechanism_Criteria == TRUE) +
    1.245*(Penetrating == TRUE) +
    0.943*(Head_Neck == TRUE) +
    0.456*(Thorax == TRUE) +
    2.776*(Pelvic == TRUE) +
    0.713*(Multiple_Regions == TRUE)
```

© 2023 Lokerman RD et al. *JAMA Netw Open.*

```
}
# Calculate linear predictors by hand
A$Linear_Predictor <- Model(
  A$Age,
  A$SPO2,
  A$GCS,
  A$Burns_Inhalation,
  A$Mechanism_Criteria,
  A$Penetrating,
  A$Head_Neck,
  A$Thorax,
  A$Pelvic,
  A$Multiple_Regions
)

# Calculate probabilities
A$Probabilities <- (1/(1+exp(-A$Linear_Predictor)))

# Calculate predictions with threshold
A$Predictions <- ifelse(A$Probabilities > 0.01450, 1, 0)
```

© 2023 Lokerman RD et al. *JAMA Netw Open.*
