# Supplementary material for: Accuracy of Prehospital Triage of Adult Patients With Traumatic Injuries Following Implementation of a Trauma Triage Intervention
Source: JAMA Netw Open. 2023 Apr 4;6(4):e236805. doi: 10.1001/jamanetworkopen.2023.6805 (PMC10074221; doi:10.1001/jamanetworkopen.2023.6805)
Supplement: Supplement 2. — Data Sharing Statement [file jamanetwopen-e236805-s002.pdf]

Data Sharing Statement

Lokerman. Accuracy of Prehospital Triage of Adult Patients With Traumatic Injuries Following Implementation of a Trauma Triage Intervention. *JAMA Netw Open*. Published April 04, 2023. doi:10.1001/jamanetworkopen.2023.6805

Data
Data available: Yes
Data types: Deidentified participant data, Other (please specify)
Additional Information: The used prediction model is freely accessible (Supplement 1). Upon request could collaborations be established with other researchers to update/adapt the models to regional agreements/circumstances. The corresponding author of this study could be contacted by sending an e-mail to rdlokerman@gmail.com
How to access data: Data and code are available upon a reasonable request that needs approval of the participating Emergency Medical Services and trauma regions, provided that ethical approval is sought. For data requests the corresponding author of this study could be contacted by sending an e-mail to rdlokerman@gmail.com
When available: With publication

Supporting Documents
Document types: Other (please specify)
Additional Information: Data and code are available upon a reasonable request that needs approval of the participating Emergency Medical Services and trauma regions, provided that ethical approval is sought. For data requests the corresponding author of this study could be contacted by sending an e-mail to rdlokerman@gmail.com
How to access documents: Data and code are available upon a reasonable request that needs approval of the participating Emergency Medical Services and trauma regions, provided that ethical approval is sought. For data requests the corresponding author of this study could be contacted by sending an e-mail to rdlokerman@gmail.com
When available: With publication

Additional Information
Who can access the data: Data and code are available upon a reasonable request that needs approval of the participating Emergency Medical Services and trauma regions, provided that ethical approval is sought. For data requests the corresponding author of this study could be contacted by sending an e-mail to rdlokerman@gmail.com
Types of analyses: Data and code are available upon a reasonable request that needs approval of the participating Emergency Medical Services and trauma regions, provided that ethical approval is sought. For data requests the corresponding author of this study could be contacted by sending an e-mail to rdlokerman@gmail.com
Mechanisms of data availability: Data and code are available upon a reasonable request that needs approval of the participating Emergency Medical Services and trauma regions, provided that ethical approval is sought. For data requests the corresponding author of this study could be contacted by sending an e-mail to rdlokerman@gmail.com
